# Performance of cytology and human papillomavirus testing in relation to the menstrual cycle

**DOI:** 10.1038/sj.bjc.6603151

**Published:** 2006-05-02

**Authors:** M E Sherman, J D Carreon, M Schiffman

**Affiliations:** 1 Division of Cancer Epidemiology and Genetics, The National Cancer Institute, Hormonal and Reproductive Epidemiology Branch, Rockville, MD, USA

**Keywords:** cervix, screening, cytology, human papillomavirus, menstrual cycle, epidemiology

## Abstract

Cervical smears prepared around the time of menses have been linked to unsatisfactory specimens and false negative results; however, it is unclear whether liquid-based cytology is similarly affected and data relating date of last menstrual period (LMP) to human papillomavirus (HPV) DNA testing are conflicting. Accordingly, we evaluated liquid-based cytology and HPV test results using Hybrid Capture 2 and PCR by LMP (days 0–10; 11–21; 22–28). We studied 5060 participants in ALTS, the Atypical Squamous Cells of Undetermined Significance (ASCUS) Low Grade Squamous Intraepithelial Lesion (LSIL) Triage Study. On average, women had 3.4 examinations (median 4, range 1–5) during a 2-year period of observation permitting an examination of intra-individual variation in cytology and HPV by LMP. Although uncommon, unsatisfactory cytology specimens were most likely on days 0–10. For satisfactory specimens, the frequency with which cytologic categories were reported varied by time since LMP, although differences were modest and did not affect the chance of abnormal cytology or its severity among women diagnosed with CIN2+. The frequency of positive HC2 tests did not vary with date of LMP. Among HPV infected women, independent of eventual diagnosis and the number of viral genotypes present, mid-cycle specimens yielded the highest frequency of LSIL cytologic interpretations and the highest HPV load; however, the magnitude of these effects were small. Intraindividual correlations of cytology or HPV by LMP were generally weak. We conclude that mid-cycle specimens yield slightly higher HPV DNA loads and slightly increased LSIL interpretations, but the clinical impact is marginal. Standardizing collection times would slightly improve interpretation of trends in HPV load. Finally, these data are consistent with the view that the biological properties of the HPV-infected cervix vary with the date of the LMP.

Important goals of cervical cancer research include improving detection of precancerous lesions and reducing equivocal results by employing better collection, preparation, and testing methods (Baldwin *et al*, 2003). Previous analyses of cytology results obtained with conventional smears have documented that suboptimal specimens result in increased reporting of false negative and equivocal results (Gay *et al*, 1985; Mitchell *et al*, 1990; Pairwuti, 1991; Henry and Wadehra, 1996; Ransdell *et al*, 1997; Mintzer *et al*, 1999; Boon *et al*, 2003; Nygard *et al*, 2004). Although many factors affect the quality of cervical cellular specimens, the time of sampling with respect to a woman's last menstrual period (LMP) has demonstrated importance.

Historically, clinicians have recognized that cytologic samples collected on days of active menstruation are typically bloody and often yield smears that are hypocellular, obscured, and lack endocervical cells (Vooijs *et al*, 1987). Furthermore, data demonstrating that unsatisfactory cytology specimens are associated with a higher than expected frequency of cervical intraepithelial neoplasia (CIN) and carcinoma (Ransdell *et al*, 1997; Nygard *et al*, 2004) in later follow-up, suggest that that these specimens may be linked to false negative results. However, efforts to coordinate return visits to re-screen women with unsatisfactory cytology often fail (McGarahan and Smith-McCune, 2005), and presumably, deferring screening for women who present near the time of menses would present similar problems. Given this dilemma, it is important to determine whether the advantages of liquid-based cytology methods, such as increased cellular recovery and reduction of obscuring by blood (Bernstein *et al*, 2001), eliminate the association between the performance of cytology and LMP that has been demonstrated for smears.

Similarly, the implementation of concurrent human papillomavirus (HPV) DNA and cytologic testing in some settings highlights the need to clarify inconsistencies in reported analyses assessing the performance of HPV testing in relation to LMP (Schneider *et al*, 1992; Fairley *et al*, 1994; Wheeler *et al*, 1996; Van Ham *et al*, 2002; Harper *et al*, 2003). Accordingly, we analyzed data for cytology and HPV DNA testing by LMP collected in the National Cancer Institute sponsored ASCUS LSIL Triage Study (ALTS).

## MATERIALS AND METHODS

### Subject selection

ASCUS LSIL Triage Study was a randomized clinical trial that enrolled subjects with community cytologic interpretations of ASCUS (*n*=3488) or LSIL (*n*=1572) at four clinical centres in the US (Schiffman and Adrianza, 2000). The study was approved by responsible review boards at the National Cancer Institute and participating institutions.

### Clinical procedures and pathology review

At enrollment, eligible subjects were interviewed regarding risk factors for cervical cancer and then underwent a pelvic examination, followed by collection of two cervical samples. The first specimen, collected with a Papette™ broom (Wallach Surgical, Orange, CT, USA), was placed in PreservCyt (Cytyc Corp., Boxborough, MA, USA) and used to prepare a ThinPrep (Cytyc) cytology slide and to perform the Hybrid Capture 2 (HC2, Digene Corp., Gaithersburg, MD, USA) test, which targets 13 oncogenic HPV types. The second sample, collected with a Dacron swab, was placed in Specimen Transport Medium (Digene), frozen, and later used for HPV typing by a polymerase chain reaction (PCR)-based assay.

Subjects were randomized to one of three management arms: (1) Conservative Management consisting of colposcopy referral for repeat cytology of a high-grade squamous intraepithelial lesion (HSIL); (2) HPV triage, with colposcopy referral for a positive HC2 test for oncogenic types (or repeat cytology of HSIL, which added almost no referrals); and (3) Immediate Colposcopy. Subjects who received histologic diagnoses at the Clinical Centers of cervical intraepithelial neoplasia 2 or worse (CIN2+) were treated with loop electrical excision procedure (LEEP). The follow-up protocol was identical for all women: repeat cytology every 6 months for 2 years, with colposcopy referral for Clinical Center cytology of HSIL. At the 24-month (exit) visit, all data were unmasked and reviewed for every subject, colposcopy was performed, and women with CIN2+ or persistent ASCUS or LSIL diagnosed by the Clinical Center were offered treatment with LEEP, permitting detection of histologic CIN2+ that was undetected by cytology and colposcopy. All referral community smears and enrollment ThinPreps, most follow-up ThinPreps, and all histology specimens were reviewed by a Pathology Quality Control (QC) Group to provide standardized interpretations and added subject safety (Schiffman and Adrianza, 2000).

### HPV testing

HC2 testing was performed as described elsewhere (Lorincz, 1996; Castle *et al*, 2004). Briefly, a 4 ml-aliquot of residual PreservCyt remaining after preparation of the thin-layer slide was used for HC2 testing and estimation of viral load when a single, targeted HPV type was present. The probe set includes HPVs 16, 18, 31, 33, 35, 39, 45, 51, 52, 56, 58, 59 and 68. Specimens in which the chemiluminescence equaled or exceeded that of a reference standard containing 1.0 pg ml^−1^ of HPV 16 DNA (approximately 5000 copies) were considered positive. The content of HPV DNA in specimens that tested positive (‘HPV load’) was determined as relative light units (RLU), calculated as the ratio of the signal of the specimen to that of the standard, which is linearly related to load (Sherman *et al*, 2002).

We performed HPV typing on DNA extracted from cells collected in specimen transport medium (Digene, Gaithersburg, MD, USA) using a PCR-based assay employing L1 consensus primers and PGMY09/11 amplification followed by reverse line blot hybridization as described elsewhere (Gravitt *et al*, 2000; Peyton *et al*, 2001). Although this PCR method is not necessarily more sensitive analytically than HC2 (Castle *et al*, 2004), we used the combination of PCR and HC2 results to determine the number of individual HPV types associated with HC2 positive specimens.

### Analysis

Clinical center cytology reports (available for all ThinPreps) and Pathology QC histology results (the reference standard for outcomes) were used for all analyses. For most analyses, we stratified these data in three periods, reflecting serum hormone fluctuations during a normal 28-day cycle: (1) days 1–10 (‘early,’ the period associated with lowest serum hormone levels); (2) days 11–21 (‘mid-cycle,’ corresponding to peak serum oestrogen levels); and (3) days 22–28 (‘late,’ associated with peak serum progesterone levels). For ease of interpretation of odds ratios (ORs), we selected the late period as the referent group, which generally yielded ORs ⩾1.0 for most comparisons.

Of 25 300 potential cytologic examinations that could have been performed in ALTS (five examinations of 5060 women), cytology results with associated LMP data in the range of 0–28 days were available for 15 389 interpretations. Missed visits (*n*=4098), failure to collect cytology specimens (*n*=16) and LMPs greater than day 28 day (*n*=5797) accounted for the exclusions. HC2 data were available for 14 490 of these visits.

We used general estimating equations (GEE) to take into account possible intra-individual ‘dependencies’ or ‘auto-correlation’ (SAS Version 9.0, Cary, NC, USA) because many women contributed multiple data points to the analysis (mean 3.4, s.d. 1.4). This approach provides an estimate of whether data from individual women are correlated; strong auto-correlation reduces the precision gains achieved for larger sample sizes, resulting in reduced statistical power. We present ORs with 95% confidence intervals (CI) using GEE models, although auto-correlation was generally weak and adjustment widened CI only slightly.

We assessed specimen quality by LMP by tabulating the frequency of reports of Bethesda System categories of ‘satisfactory for interpretation’ or ‘unsatisfactory’ (Solomon, 1991). Next, for satisfactory specimens, we examined the specific cytologic interpretations (Negative for Intraepithelial Lesion or Malignancy (NILM or Negative); ASCUS; LSIL, and High-grade Squamous Intraepithelial Lesion or worse (HSIL+)) by time since LMP for all women throughout the trial.

To consider how cytologic interpretations related to the most severe histologic diagnosis per woman during the entire trial, we stratified the cytologic results by whether or not a woman ever received a histologic diagnosis of CIN2+. Previous work within ALTS showed that subsequent risk of CIN2+ or CIN3+ was similar for women with a colposcopically-directed biopsy result of CIN1, a negative biopsy, or a normal colposcopic appearance, which did not prompt a biopsy (The ASCUS-LSIL Triage Study (ALTS) Group, 2003). We only considered cytologic interpretations that were given before the histologic diagnosis of CIN2+ that led to treatment, including the cytology result that prompted colposcopy referral (number of cytology results ranged from 1 to 5). Clinical center cytologic interpretations were grouped at three levels of cytologic abnormality (abnormal=ASCUS, LSIL, or HSIL+; abnormal=LSIL+; and abnormal=HSIL+). Taking into account auto-correlation by GEE modeling, we assessed whether LMP (three strata) affected the chance of an abnormal cytology result among women with prevalent or incipient CIN2+ as diagnosed by the Pathology QC group. We performed ancillary analyses in which we excluded data from the Conservative Management arm (which was insensitive) and in which we restricted subjects to women with histologic CIN3+.

To determine whether HC2 results varied by LMP, we assessed the frequency of positive HC2 results varied by LMP, and then repeated analyses specifically for ‘borderline HC2 results,’ previously defined as 0.8–1.5 pg ml^−1^ (Federschneider *et al*, 2004). Previous analyses in ALTS have demonstrated that infections with multiple oncogenic HPV types generally yield high HPV load. However, in specimens containing multiple types, HC2 testing does not permit the assessment of the contribution that each specific HPV type makes to the total load measurement (Sherman *et al*, 2003). Therefore, we explored whether the frequency of multiple infections (assessed by PCR) varied by LMP.

Subsequent analyses regarding HPV results were restricted to specimens with positive HC2 tests (pg ml^−1^⩾1.0) for which the corresponding PCR test demonstrated only one of the 13 oncogenic types included in the HC2 kit. The specimens could contain other types as well, not targeted by the assay. Of 3739 specimens that tested positive for only one of the 13 types by PCR and which were associated with suitable LMP dates, there were 3004 specimens that tested positive by HC2 for which we assessed viral load. To confirm the conclusions from this approach, we analyzed associations between LMP data and viral load <1.0 (which are considered negative for clinical purposes but may reflect rare HPV copies), and also analyzed LMP associations for specimens containing any single HPV type, including types that are not targeted by the HC2 kit. These analyses were confirmatory and are not presented.

We performed ancillary analyses for single infections with HPV 16 or 18, the genotypes that account for the majority of cervical cancer cases worldwide. Finally, we explored covariates such as age, numbers of sexual partners, parity, oral contraceptive use, and smoking, which were not informative and are not presented.

## RESULTS

### Frequency of cytology results and clinical performance by last menstrual period

Unsatisfactory cytology results comprised 25 of 5384 (0.46%) specimens collected early in the cycle, five of 6792 (0.07%) collected during mid-cycle, and eight of 3213 (0.25%) collected late in the cycle. Considering specimens collected late as the referent, the association with unsatisfactory specimens yielded an OR=1.87 (95% CI=0.88–4.43) for the early period and OR=0.30 (95% CI=0.09–0.89) for mid-cycle collections. Unsatisfactory reports were most common on days 0–4 (data not shown). Intra-individual correlation was minimal (−0.0019).

Among the 15 351 satisfactory specimens that comprised the rest of the analysis, the reported frequency of cytologic interpretations in ALTS varied significantly by LMP, although the magnitudes of absolute differences were slight (Table 1A). For example, ASCUS+ cytologic interpretations were associated with an OR=1.12 (95% CI=1.02–1.22) during the early period and an OR=1.10 (95% CI=1.01–1.20) during mid-cycle, compared with the late period. A minimally elevated percentage of LSIL+ (vs⩽ASCUS) was found for mid-cycle specimens (OR=1.14, 95% CI=1.03–1.28) compared with the late period. Reports of HSIL declined progressively in frequency from early to late in the cycle, yielding an OR=1.38 (95% CI=1.10–1.75) for the early period and an OR=1.20 (95% CI=0.96–1.51) for mid-cycle. Of note, at any level of severity, the intra-individual correlation of cytologic interpretations for multiple specimens per woman was weak (<0.25).

To explore the possible clinical relevance of the variation in cytology by time since LMP, we restricted the study population to women who received a histologic diagnosis of CIN2+ in ALTS (Table 1B). Among these women with prevalent or incipient histologic CIN2+, cytology of ASCUS+ during the three LMP intervals ranged from 82.4% (early cycle) to 79.8% (mid-cycle) to 82.8% (late cycle). The corresponding ORs were nonsignificant. Similarly, we did not find substantial heterogeneity for cytology of LSIL+ (vs ASCUS or negative) or HSIL+ (vs LSIL or less severe). Analyses limited to women diagnosed with histologic CIN3+, and those restricted to the HPV- and IC-Arms ALTS yielded similar results (data not shown).

### HPV testing results stratified by last menstrual period and other factors

We compared the frequency of HPV DNA positive test results by HC2 for the three intervals of the menstrual cycle, without restrictions. The auto-correlation estimate was 0.39, showing a slight tendency of women that were HPV positive once to be positive repeatedly. This strength of correlation was typical of all HPV analyses, whether assessed as negative vs positive or as viral load.

Overall, 44.6% of specimens were HC2 positive. Frequencies of HC2 positive results were unrelated to the date of LMP. When we assessed viral load among all HC2-positive tests (*n*=6468 associated with one or multiple types by PCR), we obtained slightly significant associations. Compared to specimens collected late in the cycle, specimens obtained early in the cycle had viral loads that were only 83% (95% CI=0.71–0.98) as high, whereas mid-cycle specimens yielded average loads that were 22% higher (95% CI=1.05–1.43).

The frequency of ‘borderline’ positive HC2 results varied only slightly by date of LMP: 4.4% early in the cycle, 3.8% mid-cycle, and 3.9% late in the cycle. These differences were not significantly different.

Overall, positive HC2 test results and high viral loads are more common for specimens containing multiple HPV types. Therefore, we separately assessed the influence of LMP on load per type and on the detection of multiple types. Date of LMP was not associated with detection of multiple types by PCR (data not shown).

To study the associations between HPV viral load per type and time of LMP, independent of number of HPV types present, we assessed the frequency of positive HC2 results among specimens associated with PCR detection of one of the 13 carcinogenic types targeted by HC2. Positive HC 2 results were obtained for 1094 of 1377 (79.4%) specimens collected early in the cycle, 1315 of 1614 (81.5%) obtained at mid-cycle, and 595 of 748 (79.6%) late cycle specimens (Table 2). Compared with specimens collected late in the cycle, the ORs for HC2 positive tests were 0.99 (95% CI=0.80–1.24) early in the cycle and 1.13 (95% CI=0.91–1.40) mid-cycle, demonstrating a lack of a statistically significant association.

**Figure 1:**
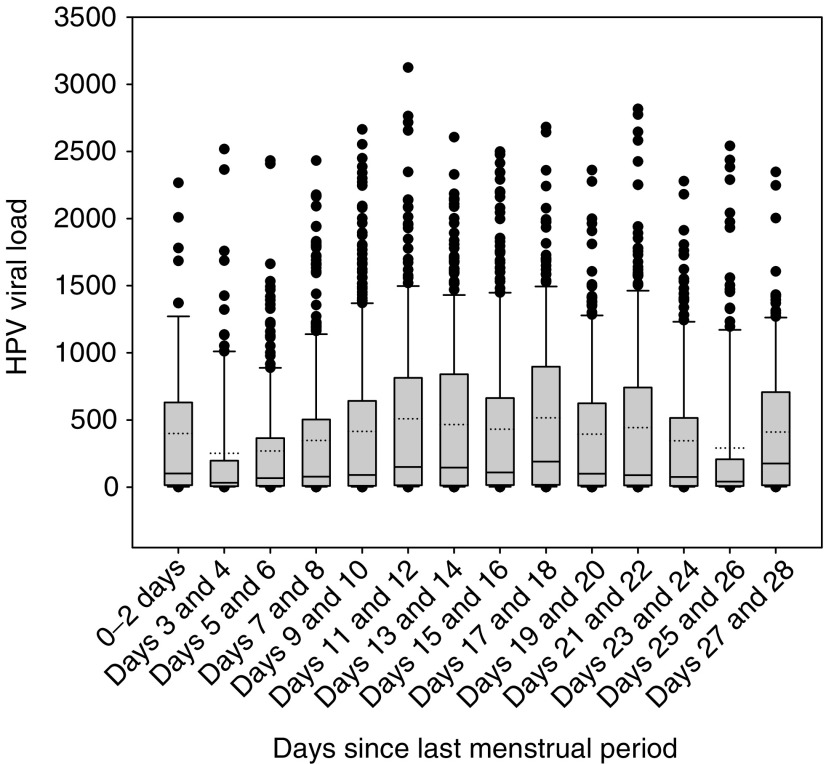
Human papillomavirus load vs days since last menstrual period in 2-day intervals for all Hybrid Capture 2 positive results (pgml^−1^⩾1.0). The analysis is restricted to specimens associated with only one of the 13 carcinogenic types targeted by HC2, as determined by a PCR-based assay performed on a second concurrently collected specimen.

However, among HC2 positive specimens (RLU⩾1.0) containing only one of the 13 types, HPV load (overall mean pg ml^−1^=403.1, median 93.6) peaked at mid-cycle (Figure 1 and Table 2). The general pattern of HPV load was a gradual increase from days 3 to 4 to reach a plateau at mid-cycle, followed by a decline in the late luteal phase. Among samples containing a single type based on the PCR assay, those collected at mid-cycle demonstrated 42% higher viral loads by HC2 (95% CI=1.14–1.77) than those collected late in the cycle. Tests performed on specimens collected between days 0–2 and days 27–28 demonstrated higher values than specimens obtained during flanking intervals.

### Joint consideration of cytology and HPV test results by LMP

Increased viral load and cytologic abnormality were tightly linked, making it impossible to determine which component was more strongly associated with LMP. As shown in Table 2, mid-cycle specimens showing SIL (with one of the 13 types) had a viral load that was 38% higher on average (95% CI=1.03–1.83) compared with those collected late in the cycle. The increase in viral load was weaker for ASCUS, and lacking when cytology was Negative. Among HC2 positive specimens, the ratio of LSIL to ASCUS was highest at mid-cycle, either as a cause or result of higher viral load. The frequency of HPV-negative ASCUS+ (mainly ASCUS because HPV-negative SIL is rare) did not vary by time of menstrual cycle (data not shown). In sum, at mid-cycle viral load was higher when cytology was abnormal, and cytology looked more definitively abnormal when specimens were HPV-positive (Table 3)

Finally, we analysed data for single infections with HPV16 (*n*=468 infected specimens) and HPV18 (*n*=192) separately. Among specimens collected early in the cycle that were associated with concurrent single infections with HPV 16 (determined by PCR), 77.7% of HC2 tests were positive, which was less sensitive than HC2 tests performed on specimens collected late in the menstrual cycle (OR=0.52, 95% CI=0.26–0.98). Viral load was nonsignificantly lower (21.9%) for the specimens collected early as opposed to late in the cycle. For mid-cycle specimens associated with HPV 16, the frequency of HC2 positive results and load determinations did not differ significantly from those obtained for specimens late in the cycle. Among specimens associated with concurrent detection of single infections with HPV 18 (detected by PCR), positive HC2 results were more frequent at mid-cycle (OR=2.83, 95% CI=1.09–7.39) and load was 55% higher (though non-significantly) compared to specimens collected late in the cycle.

## DISCUSSION

This study demonstrates that the performance of thin-layer cytology and HPV testing varies during the menstrual cycle, although the fluctuations are modest. Cervical specimens collected at mid-cycle (days 11–21) are probably optimal for detection of HPV DNA by HC2 testing and for identification of LSIL by thin-layer cytology. However, the effects of the date of LMP on the performance of cytology and HC2 testing among women diagnosed with histologic CIN2+ was minor, suggesting that it is unnecessary to defer testing for women who present outside this interval.

Ideally, optimizing the timing of specimen collection would minimize unsatisfactory specimens and cytologic reporting of ASCUS (now called ASC) overall and maximize clear-cut results of SIL among women with underlying CIN2+. Similar to results obtained with conventional smears, specimens obtained early in the menstrual cycle were associated with the highest frequency of unsatisfactory samples (Vooijs *et al*, 1987). Although addition of glacial acetic acid to bloody cervical specimens may reduce the frequency of unsatisfactory thin-layer slides, data suggest that this treatment may cause false positive HC2 results, thus limiting the subsequent utility of such specimens (Agoff *et al*, 2002).

Specimens collected early in the cycle yielded the high frequency of HSIL cytology overall and among women who were diagnosed with histologic CIN2+, which could reflect improved sensitivity of these samples compared to those obtained at other times in the cycle. However, it is notable that reporting of HSIL+ cytology was also highest early in the cycle among women who were not diagnosed with histologic CIN2+. In addition, HPV negative test results associated with HSIL+ cytology were slightly higher early in the cycle (7.3%) as compared to mid-cycle (4.4%) and late in the cycle (2.9%), although these differences were not significantly significant based on limited data. To account for these findings, we speculate that misclassification of endometrial cells as HSIL contributes to higher reporting of HSIL during the early part of the cycle. In support of this hypothesis: (1) endometrial cells are generally identified only during the first 10 days of the cycle; (2) endometrial cells may mimic HSIL because of their similar small size and high nuclear to cytoplasmic ratio, especially in liquid-based cytology; and (3) the inconspicuousness of blood in most thin-layer slides disguises the occurrence of menses. Previous studies have emphasized that interpretation of HSIL in thin-layer cytology may pose difficulties, especially with regard to false negative results (Wilbur *et al*, 1996; Renshaw *et al*, 2004). Given that both false negative and false positive interpretations of HSIL may have undesirable consequences, efforts to improve the recognition of HSIL are warranted.

Published data related to whether HPV detection varies with time since LMP are inconclusive. In this analysis, the frequency of positive HC2 results varied little by date of LMP. In a study based on PCR testing of tampon specimens, HPV detection was also unrelated to quartiles of the menstrual cycle (Fairley *et al*, 1994). Two studies in which subjects were repeatedly tested using PCR based assays found null associations between HPV detection and LMP; however, these reports conflicted about whether recent intercourse was associated with positive test results (Wheeler *et al*, 1996; Harper *et al*, 2003). Other reports based on repeated testing have found associations between HPV detection and LMP. One study demonstrated increased detection of HPV 16 during the luteal phase (Schneider *et al*, 1992) and another identified HPV more often between days 7 and 11 of the menstrual cycle (Van Ham *et al*, 2002). Although the relationship between HPV detection and LMP remains unclear, studies have consistently found that point prevalence dramatically under-estimates cumulative prevalence, indicating that low load infections are missed with one-time testing.

Although the frequency of positive HC2 results among women with a single carcinogenic infection was not associated with time since LMP, we did find that HPV load among positive specimens was modestly increased at mid-cycle, particularly among women with cytology of SIL. These data are consistent with the increase in abnormal cytology, particularly LSIL, at mid-cycle. The strong correlation between cytology and load precludes a clear determination of whether the main effect of LMP is to increase HPV load or evoke more abnormal cytology interpretations. Given either interpretation, these findings suggest that adjusting for time since LMP or collecting samples within the same phase of the cycle may improve the interpretation of trends in viral load based on repeated testing. Although HPV load measurements are too variable to be used routinely for clinical management (Sherman *et al*, 2002, 2003) data are conflicting about whether determining HPV 16 load has value in identifying women at elevated risk for developing cervical neoplasia in later follow-up (Josefsson *et al*, 2000; Lorincz *et al*, 2002). It is interesting that HC2 results were less likely to be positive early in the cycle for women infected with HPV 16 and that women with HPV 18 infections were more likely to test HC2 positive at mid-cycle, although the underlying reasons are unknown.

The mechanisms that result in measurement of higher HPV loads at mid-cycle are obscure. We hypothesize that the peak oestrogen levels at mid-cycle promote this effect by reducing cellular adhesion, or by enhancing HPV viral replication, suppressing local immunity or other alterations. In both animal models and humans, oestrogen promotes epithelial hyperplasia, metaplasia, and maturation of cervical epithelium, creating a microenvironment suitable for HPV replication (Arbeit *et al*, 1996; Elson *et al*, 2000). In HPV 16 transgenic mice, estrogen treatment is required for carcinogenesis (Elson *et al*, 2000; Brake and Lambert, 2005). In cell cultures, oestrogen interacts with adhesion molecules, producing increased deformability and contraction, which theoretically could facilitate cellular exfoliation (Gorodeski, 1998). Finally, levels of immunoglobulins in cervical secretions reach their nadir at mid-cycle, which may favour HPV replication and accumulation to high viral loads (Franklin and Kutteh, 1999; Nardelli-Haefliger *et al*, 2003). The explanation for the observed, more minor spike in viral load around the time of menses is also unexplained, although apoptosis secondary to oestrogen withdrawal represents a possible explanation (Wang *et al*, 2004).

Although this analysis was based on a clinical trial of over 5000 women who were followed with repeated examinations for 2 years, we recognize some limitations. The majority of women in this study were young and eligibility criteria included a recent cytology report of ASCUS or LSIL, therefore, these results may not apply to all women. In addition, the measurement of HPV load in cervical cellular collections using HC2 does not necessarily reflect the concentration of the virus in cervical tissue. In fact, it is recognized that HC2 pg ml^−1^ values <1.0 may represent low levels of infection; the analytical cutpoint for HC2 assays was selected to optimize clinical sensitivity (i.e. disease detection) rather than analytical sensitivity (detection of the lowest number of copies possible). Finally, some collections that yielded positive PCR and negative HC2 tests could reflect errors in HPV typing, differences in HPV content between samples tested by HC2 and PCR or other factors.

In conclusion, the LMP date is not a critical clinical consideration if women with cytology of ASCUS+ are closely monitored and HPV test results are categorized as negative or positive for management. However, it is prudent when possible to schedule screening tests at mid-cycle to optimize sensitivity and limit the occurrence of unsatisfactory cytology. Standardized timing of specimen collections may also minimize the inherent variability of test results for individual patients, thereby facilitating interpretation of serial results. Improved understanding of the interactions between hormones and HPV may eventually provide clues that are useful for cancer prevention.

## Figures and Tables

**Table 1A:** Frequency (%) of cytologic interpretations stratified by last menstrual period (LMP)

	**All cytologic results during 2-year period of trial**			
**LMP – days**	**Negative**	**ASCUS**	**LSIL**	**HSIL+**
0–10 (*n*=5359)	3008 (56.1)	1373 (25.6)	734 (13.7)	244 (4.6)
11–21 (*n*=6787)	3839 (56.6)	1597 (23.5)	1081 (15.9)	270 (4.0)
22–28 (*n*=3205)	1886 (58.9)	747 (23.3)	465 (14.5)	107 (3.3)

**Table 1B:** Frequency (%) of cytologic interpretations stratified by last menstrual period (LMP), among women diagnosed with CIN2+

	**All cytologic results during 2-year period preceding CIN2+**			
**LMP – days**	**Negative**	**ASCUS**	**LSIL**	**HSIL+**
0–10 (*n*=499)	88 (17.6)	145 (29.1)	130 (26.1)	136 (27.3)
11–21 (*n*=618)	125 (20.2)	140 (22.7)	201 (32.5)	152 (24.6)
22–28 (*n*=308)	53 (17.2)	76 (24.7)	107 (34.7)	72 (23.4)

**Table 2:** Positive HC tests and HPV load by last menstrual period (LMP), overall and stratified by cytology

	**HC2 positive results among women with single infections^a^**				**Viral load of HC2 positive specimens, associated with single infections^a^**				
	**% HC2+**	**0–10 days OR (95%CI)**	**11–21 days OR (95%CI)**	**22–28 days**	**Mean pg/ml**	**Median pg/ml**	**0–10 days OR (95%CI)**	**11–21 days OR (95%CI)**	**22–28 days**
All samples (*n*=3739)	80.3	0.99 (0.80–1.24)	1.13 (0.91–1.40)	1 (ref)	403.11	93.62	0.96 (0.76–1.20)	**1.42** (**1.14–1.77)**	1 (ref)
SIL (*n*=1186)	96.8	0.83 (0.31–1.97)	1.24 (0.47–2.98)	1 (ref)	721.0	533.86	0.90 (0.67–1.22)	**1.38** (**1.03–1.83)**	1 (ref)
ASCUS (*n*=981)	85.4	1.23 (0.76–1.98)	1.18 (0.73–1.87)	1 (ref)	323.4	75.07	0.84 (0.56–1.26)	1.16 (0.78–1.74)	1 (ref)
Negative (*n*=1566)	64.8	0.87 (0.65–1.15)	0.93 (0.70–1.22)	1 (ref)	110.6	14.89	0.97 (0.70–1.33)	1.01 (0.74–1.38)	1 (ref)

a One of 13 types of carcinogenic HPV targeted by HC2, as detected by PCR; cytology interpretations were unsatisfactory for six samples.

Statistically significant values in bold.

**Table 3:**
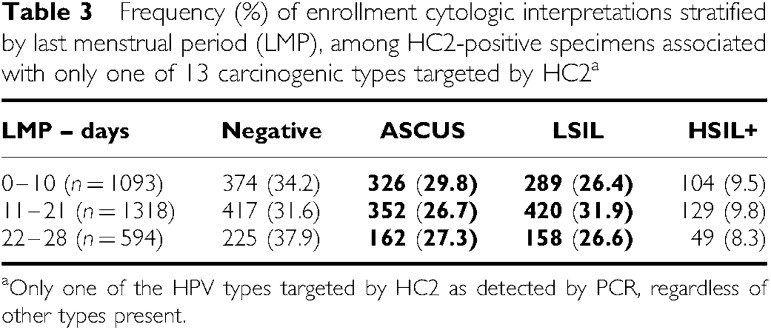
Frequency (%) of enrollment cytologic interpretations stratified by last menstrual period (LMP), among HC2-positive specimens associated with only one of 13 carcinogenic types targeted by HC2^a^

## **Appendix A.**

### AFFILIATIONS OF THE ALTS GROUP

National Cancer Institute, Bethesda, MD, USA: D Solomon, Project Officer M Schiffman, Co-Project Officer Clinical Centers: University of Alabama at Birmingham, AL, USA: EE Partridge, Principal Investigator L Kilgore, Co-Principal Investigator S Hester, Study Manager University of Oklahoma, Oklahoma City, OK, USA: JL Walker, Principal Investigator GA Johnson, Co-Principal Investigator A Yadack, Study Manager Magee-Womens Hospital of the University of Pittsburgh Medical Center Health System, Pittsburgh, PA, USA: RS Guido, Principal Investigator K McIntyre-Seltman, Co-Principal Investigator RP Edwards, Investigator J Gruss, Study Manager University of Washington, Seattle, WA, USA: NB Kiviat, Co-Principal Investigator L Koutsky, Co-Principal Investigator C Mao, Investigator Colposcopy Quality Control Group: D Ferris, Principal Investigator, Medical College of Georgia, Augusta, GA, USA JT Cox, Co-Investigator, University of California at Santa Barbara, Santa Barbara, CA, USA L Burke, Co-Investigator, Beth Israel Deaconess Medical Center Hospital, Boston, MA, USA HPV Quality Control Group: CM Wheeler, Principal Investigator, University of New Mexico Health Sciences Center, Albuquerque, NM, USA C Peyton-Goodall, Lab Manager, University of New Mexico Health Sciences Center, Albuquerque, NM, USA MM Manos, Co-Investigator, Kaiser Permanente, Oakland, CA, USA Pathology Quality Control Group: RJ Kurman, Principal Investigator, Johns Hopkins Hospital, Baltimore, MD, USA DL Rosenthal, Co-Investigator, Johns Hopkins Hospital, Baltimore, MD, USA ME Sherman, Co-Investigator, Johns Hopkins Hospital, Baltimore, MD, USA MH Stoler, Co-Investigator, University of Virginia Health Science Center, Charlottesville, VA, USA Quality of Life Group: DM Harper, Investigator, Dartmouth Hitchcock Medical Center, Lebanon, NH, USA Westat, Coordinating Unit, Rockville, MD, USA J Rosenthal, Project Director M Dunn, Data Management Team Leader J Quarantillo, Senior Systems Analyst D Robinson, Clinical Center Coordinator Digene Corporation, Gaithersburg, MD, USA: A Lorincz, Senior Scientific Officer Information Management Services Inc., Silver Spring, MD, USA: B Kramer, Senior Programmer/Analyst.
